# Quantifying the Clinical Significance of Cannabis Withdrawal

**DOI:** 10.1371/journal.pone.0044864

**Published:** 2012-09-26

**Authors:** David J. Allsop, Jan Copeland, Melissa M. Norberg, Shanlin Fu, Anna Molnar, John Lewis, Alan J. Budney

**Affiliations:** 1 National Cannabis Prevention and Information Centre, National Drug and Alcohol Research Centre, University of New South Wales, Sydney, New South Wales, Australia; 2 Centre for Forensic Science, School of Chemistry and Forensic Science, University of Technology Sydney (UTS), New South Wales, Australia; 3 Geisel School of Medicine at Dartmouth, Hanover, Lebanon, New Hampshire, United States of America; University of Granada, Spain

## Abstract

**Background and Aims:**

Questions over the clinical significance of cannabis withdrawal have hindered its inclusion as a discrete cannabis induced psychiatric condition in the Diagnostic and Statistical Manual of Mental Disorders (DSM IV). This study aims to quantify functional impairment to normal daily activities from cannabis withdrawal, and looks at the factors predicting functional impairment. In addition the study tests the influence of functional impairment from cannabis withdrawal on cannabis use during and after an abstinence attempt.

**Methods and Results:**

A volunteer sample of 49 non-treatment seeking cannabis users who met DSM-IV criteria for dependence provided daily withdrawal-related functional impairment scores during a one-week baseline phase and two weeks of monitored abstinence from cannabis with a one month follow up. Functional impairment from withdrawal symptoms was strongly associated with symptom severity (p = 0.0001). Participants with more severe cannabis dependence before the abstinence attempt reported greater functional impairment from cannabis withdrawal (p = 0.03). Relapse to cannabis use during the abstinence period was associated with greater functional impairment from a subset of withdrawal symptoms in high dependence users. Higher levels of functional impairment during the abstinence attempt predicted higher levels of cannabis use at one month follow up (p = 0.001).

**Conclusions:**

Cannabis withdrawal is clinically significant because it is associated with functional impairment to normal daily activities, as well as relapse to cannabis use. Sample size in the relapse group was small and the use of a non-treatment seeking population requires findings to be replicated in clinical samples. Tailoring treatments to target withdrawal symptoms contributing to functional impairment during a quit attempt may improve treatment outcomes.

## Introduction

The Diagnostic and Statistical Manual of Mental Disorders (DSM-IV) requires that a mental health diagnosis “..causes clinically significant distress or impairment in social, occupational, or other important areas of functioning” ([1], p.358) in order to reduce false positive diagnoses (i.e. incorrectly labelling somebody with a mental health disorder). For DSM-IV drug dependence, at least three of the following seven diagnostic markers must cause clinically significant functional impairment ([1], p.181–183): *1. tolerance to the substance, 2. consumption in larger amounts or for longer periods than intended, 3. a persistent desire or unsuccessful attempts to cut down, 4. a great deal of time spent obtaining, using or recovering from the substance, 5. important activities are given up or reduced because of the substance, 6. substance use is continued despite the knowledge that it causes problems, 7. the presence of characteristic withdrawal symptoms or use of substance to alleviate withdrawal.* Cannabis however, unlike other drugs, does not currently include the seventh criterion of withdrawal for diagnosing a cannabis use disorder in the DSM-IV. This is due to debate about the clinical significance of the cannabis withdrawal syndrome.

The evidence-base for cannabis withdrawal [2], [3], [4], [5], [6], [7] has led to a proposal to include it in the DSM-5 (see and [8], [9]), which could increase the prevalence of cannabis dependence diagnoses in the community [10]. Increases in the prevalence of any mental health disorder can have ramifications for treatment service provision, highlighting the importance of ensuring that cannabis withdrawal is clinically significant. To address this, a valid and reliable Cannabis Withdrawal Scale (CWS) is in the early phases of development, and the initial study validated the CWS via self-ratings of the intensity of withdrawal symptoms during cannabis abstinence [11]. While measurement of symptom intensity *per se* is a central tenet of clinical scales of alcohol and other drug withdrawal to date [12], [13], [14], [15], [16], intensity measures do not necessarily capture the clinical significance associated with each symptom or with the syndrome as a whole. In addition to measuring the intensity of withdrawal symptoms, a more direct method to assess their clinical significance would draw on the DSM definition, and explicitly quantify how much symptoms impair normal daily functioning such as required for work, family life, and social functioning.

Research attempting to demonstrate the clinical significance of cannabis withdrawal has used two approaches: (a) linking withdrawal intensity to distress and/or substance use [5], [6], [17], [18], [19], [20], [21], [22], and (b) demonstrating that cannabis withdrawal is of a similar magnitude and has similar consequences to nicotine withdrawal, a well accepted clinically valid syndrome [20], [23], [24]. In regards to linking withdrawal symptoms to cannabis use, two retrospective studies showed that craving was the most highly endorsed withdrawal symptom by people who relapsed, followed by irritability, anger and boredom [4], [5]. However the use of only relapse as a measure of clinical significance may mask the extent to which symptoms led to functional impairment, as those who maintained abstinence may still have experienced clinically significant negative consequences from cannabis withdrawal (e.g. relationship or work problems resulting from the withdrawal syndrome).

Two studies have looked at the clinical significance of individual cannabis withdrawal symptoms using Likert scales to tease apart variation in the level of functional impairment. In a retrospective survey of adults who made a recent quit attempt, Budney et al. (2008) [20] used a 10-point Likert scale to show that the *intensity* of aggression, anger, anxiety, cravings, and depression symptoms contributed to cannabis relapse. Allsop and colleagues [11] used a 10-point Likert scale in a prospective study using a nonclinical outpatient population to measure withdrawal symptom intensity as well as the functional impairment caused by each symptom. The items causing the most impairment to normal daily functioning were: trouble getting to sleep, angry outbursts, imagining being stoned (cravings), loss of appetite, feeling easily irritated, and nightmares or strange dreams. The present study extends that work by exploring whether the functional impairment reported during abstinence is clinically significant, and what factors predict it.

This study tested in a non clinical sample of non-treatment seekers, (1) whether the level of functional impairment during abstinence is predicted by severity of dependence, or pre-quit attempt cannabis use levels, whilst controlling for age and gender, and (2) what the relationship is between the intensity of cannabis withdrawal symptoms and the level of associated functional impairment. In addition the study had the following exploratory aims: (a) to test the hypothesis that relapse to cannabis use is associated with greater levels of functional impairment from cannabis withdrawal symptoms, (b) to test the hypothesis that greater functional impairment during the abstinence attempt is predictive of a greater amount of cannabis consumed during a one month follow-up period, and (c) to test what factors predict time to relapse.

## Methods

### Participants

Current cannabis users who were not seeking treatment for their cannabis use were recruited from Sydney, Australia using a targeted postcard campaign (http://www.webcitation.org/69yO6gGfy) and advertisements in local newspapers asking for people who were prepared to abstain from cannabis for a two-week period for research purposes. Inclusion criteria included: (a) cannabis use on five or more days per week over the previous three months; (b) current cannabis dependence; (c) previous experience of at least one cannabis withdrawal symptom; and (d) willingness to quit cannabis for two weeks. Exclusion criteria included: (a) moderate or severe dependence on other substances except caffeine and nicotine; (b) substance-related treatment in the last three months; and (c) pregnancy or planning on becoming pregnant during the study. After a complete description of the study to the participants, written informed consent was obtained.

### Measurements

A phone screening interview was used to collect demographics, cannabis dependence severity using the Severity of Dependence Scale (SDS) [25], [26], and hazardous alcohol consumption using the Alcohol Use Disorders Identification Test (AUDIT) [27]. The SDS contains five items and uses a four-point response scale and is reported to have high internal consistency (Chronbach's alpha = 0.83, high test-retest reliability (Interclass Correlation Coefficient (ICC) = 0.88), and good concurrent validity [26], [28]. The AUDIT is a 10-item questionnaire developed by the World Health Organisation, each item is scored on a four-point scale, and different question groups measure hazardous consumption or dependence behaviour. The AUDIT's psychometric properties have been demonstrated to be excellent in a wide range of studies, with high internal consistency (Chronbach's alpha ∼0.83), test-retest reliability (ICC = 0.87–0.93) and good concurrent validity [29]. Telephone administration has proved efficient and successful for both the AUDIT [30], and the SDS [33], [34]. The Structured Clinical Interview for DSM Disorders-Research Version (SCID-RV) [35] was administered at the baseline laboratory visit by a trained psychologist to assess for Axis-I psychiatric disorders. The Timeline Followback (TLFB) [36], [37] was used to assess alcohol, tobacco and cannabis use at each laboratory visit. The major urinary metabolite of cannabis, 11-nor-Δ^9^-tetrahydrocannabinol-9-carboxylic acid (THC-COOH) was quantified by gas chromatography-mass spectrometry and normalized by urinary creatinine level (THC-COOH/creatinine) to validate self reported abstinence [38].

An online version of the CWS [11], including a functional impairment subscale, was used to quantify the impact of cannabis withdrawal symptoms on normal daily functioning using a 10-point Likert scale question asking how each symptom NEGATIVELY impacted getting through or completing normal daily activities, assessed alongside withdrawal intensity (see Table 1). The CWS has been shown to have excellent psychometric properties, with high internal reliability (Chronbach's alpha = 0.91) and test-retest reliability (ICC = 0.95) [11]. Whilst the withdrawal symptoms on the CWS do not represent functional impairment *per se* (e.g. not being able to socialise), the negative impact component on the CWS anchored to each symptom specifically addresses this question by having patients give an indication of the magnitude of impairment to normal daily functioning caused by each symptom.

**Table 1:** The cannabis withdrawal scale.

Not at all Moderately Extremely													Negative Impact on daily activity (0–10)
**1**	The only thing I could think about was smoking some cannabis	0	1	2	3	4	5	6	7	8	9	10	
**2**	I had a headache	0	1	2	3	4	5	6	7	8	9	10	
**3**	I had no appetite	0	1	2	3	4	5	6	7	8	9	10	
**4**	I felt nauseous (like vomiting)	0	1	2	3	4	5	6	7	8	9	10	
**5**	I felt nervous	0	1	2	3	4	5	6	7	8	9	10	
**6**	I had some angry outbursts	0	1	2	3	4	5	6	7	8	9	10	
**7**	I had mood swings	0	1	2	3	4	5	6	7	8	9	10	
**8**	I felt depressed	0	1	2	3	4	5	6	7	8	9	10	
**9**	I was easily irritated	0	1	2	3	4	5	6	7	8	9	10	
**10**	I had been imagining being stoned	0	1	2	3	4	5	6	7	8	9	10	
**11**	I felt restless	0	1	2	3	4	5	6	7	8	9	10	
**12**	I woke up early	0	1	2	3	4	5	6	7	8	9	10	
**13**	I had a stomach ache	0	1	2	3	4	5	6	7	8	9	10	
**14**	I had nightmares and/or strange dreams	0	1	2	3	4	5	6	7	8	9	10	
**15**	Life seemed like an uphill struggle	0	1	2	3	4	5	6	7	8	9	10	
**16**	I woke up sweating at night	0	1	2	3	4	5	6	7	8	9	10	
**17**	I had trouble getting to sleep at night	0	1	2	3	4	5	6	7	8	9	10	
**18**	I felt physically tense	0	1	2	3	4	5	6	7	8	9	10	
**19**	I had hot flashes	0	1	2	3	4	5	6	7	8	9	10	

**Instructions:** This version of the CWS asks about symptoms experienced over the last 24 hours, and can be administered by an interviewer OR by self report.

The following statements describe how you have felt over the last 24 hours. Please **circle the number** that most closely represents your personal experiences for each statement. For each statement, please rate its negative impact on normal daily activities on the same scale (0 = Not at all to 10 = Extremely), writing the number in the right hand column.

Score by summing each items value to a maximum withdrawal score of 190 (you can derive two scores from the scale: one for withdrawal intensity and one for the negative impact of withdrawal – each separate score has a theoretical maximum of 190).

Reprinted from Drug and Alcohol Dependence, Vol 119, Allsop, D.J., Norberg, M.M., Copeland, J., Fu, S., Budney, A.J. The Cannabis Withdrawal Scale development: Patterns and predictors of cannabis withdrawal and distress, 123–129., Copyright (2011), with permission from Elsevier (License number 2872801116106).

### Study procedures and cannabis use

The University of New South Wales Human Research Ethics Committee approved all procedures (Approval number: HREC 09152). Study participants filled out the CWS online daily during a one-week baseline “smoking as usual” period, and a two-week cannabis abstinence attempt. The cannabis abstinence attempt was supported with a one-hour psychological intervention and contingency management payments totalling AU$450 for adherence to study protocol, including the provision of urine samples indicating no cannabis use during the two week abstinence period. Participants in the study visited the research facility five times over the course of their involvement in the study: once at baseline, once after a week of smoking as usual, once after the first week of abstinence, and again at the end of the second week of abstinence. A final visit for follow-up interviews was performed one month after the end of the experimental abstinence period. Study procedures, including eligibility screening, and a full documentation of face to face interview schedules, the content of the 1 hour psychological intervention at the beginning of the quit attempt and monitoring of study adherence (including confirmation of cannabis abstinence) are described in a previous report [11]. If participants used cannabis in the first abstinence week, they were offered an opportunity to restart the abstinence period. If they restarted, data from their first abstinence attempt, up to the day of cannabis use, is used in the current functional impairment analysis. If participants used cannabis during the second week of abstinence, any post cannabis use withdrawal data were discarded and all functional impairment data collected prior to cannabis use was retained for analysis. Participants were considered to have used cannabis if they self-reported cannabis use or if their THC-COOH∶creatinine ratios showed any increase during the abstinence phase relative to their one week ‘smoking as usual’ baseline phase levels [39]. Data from two participants were removed from all analyses due to a conflict between their self-reported cannabis use and urinalysis tests at weeks 1 and 2 of the abstinence period. All other participants' cannabis abstinence reports were validated by urinalysis. Participant's cannabis use during the month following the end of their abstinence period was monitored by self report (TLFB) and confirmed with a single urinalysis at the one month follow up interview requiring THC-COOH∶creatinine levels to be below 50 ng/ml to be classed as abstinent.

### Analysis

Descriptive statistics were reported as frequency and means with standard deviations and ranges (except where non-parametric analyses were performed, where continuous variables were described using medians and interquartile range). Analysis of Variance, Pearson Chi Square and Fishers exact test were used to compare clinical characteristics of: (1) participants who relapsed to those who didn't relapse, and (2) participants who were lost to follow up to those who were not lost to follow-up.

### Aim 1: Identify if the level of functional impairment during abstinence is predicted by severity of dependence, or pre-quit attempt cannabis use levels, whilst controlling for age and gender

To explore whether the level of functional impairment could be predicted by cannabis use, severity of dependence, age or gender, a General Linear Mixed Model (GLMM) was constructed with total daily functional impairment scores (summed across all 19 valid items in a day) as the dependent variable. The dependent variable represents repeated measurements, and the GLMM allows explicit modelling of covariance between daily measures within individual subjects (using an autoregressive covariance structure of order 1). The model was constructed in a hierarchical manner, with the null model consisting of the intercept only. Step one explored the effect of time in abstinence on functional impairment scores, as abstinence represents the primary and most fundamental independent variable generating withdrawal phenomena. Step two added the non-cannabis use related covariates (age and gender) in order to ensure they are controlled for ahead of adding the cannabis use variables, which are the hypothesised drivers of withdrawal related functional impairment. Step three added cannabis-related variables (pre-quit cannabis use levels and scores on the SDS), to test their relative explanatory power having controlled for other variables. SDS scores were analysed as continuous variables as dichotomising loses valuable statistical information and power and obscures any nonlinearities between variables [40], [41]. However for graphical purposes the data were split into high and low SDS groups.

As mixed-effects models do not generate traditional R^2^ values, the variance in withdrawal related functional impairment explained by the variables at each step of the model was estimated using a pseudo R^2^ calculated from the log likelihood ratios output from mixed models (termed R^2^_LR_) [42]. Because R^2^ values are known to increase with the number of variables in a model, irrespective of their predictive power, the model also presents Akaikes Information Criteria [43] as a measure of model fit for each step, as this value penalises models for increased complexity. Because of sample size restrictions the analysis was not powered to look at the interactions in this longitudinal analysis.

### Aim 2: Identify if a relationship exists between withdrawal related functional impairment and the severity of cannabis withdrawal symptoms

In order to examine the relationship between withdrawal severity and functional impairment, it was determined that all other possible drivers of functional impairment should first be controlled for. Hence the full model from Aim 1, examining the predictors of functional impairment was retained, with the addition of a final step. In this final step, the effect of adding CWS symptom severity scores on the explained observed variance in functional impairment was examined.

### Exploratory Aim 1: Relapse to cannabis use is associated with greater levels of functional impairment from cannabis withdrawal symptoms

To assess if participants who relapsed during the abstinence period had higher levels of impairment from cannabis withdrawal, each symptom's functional impairment score was analysed separately using a univariate approach. Rank transformed functional impairment scores were used as dependent variables in a series of non parametric two way repeated measures Analysis of Variance [44], with time as the repeated measure (baseline week vs. abstinence) and relapse group as the between subject factor. Symptoms were then sorted (separately for each SDS group [45]) on their univariate F-values for the interaction between time and relapse group.

Withdrawal symptoms significant in the univariate analyses were then entered as independent variables in a multivariate logistic regression [44] by subtracting impairment scores from abstinence week 1 from baseline smoking as usual scores to create ‘change’ variables. Membership of the relapse group (or not) was the binary dependent variable. To fully explore the withdrawal parameter space contributing to relapse, the selected withdrawal symptoms were grouped into either somatic or negative affect symptoms.

### Exploratory Aim 2: Greater functional impairment during the abstinence attempt is predictive of a greater amount of cannabis consumed during a one-month follow-up period

To test the impact of functional impairment during abstinence on levels of cannabis use at one-month follow up, a linear regression (Generalized Linear Model – GLM) was constructed with average weekly cannabis use at follow-up as the dependent variable. The independent predictor was the CWS sum total functional impairment score, calculated by averaging daily scores across the two-week abstinence period. Pre-study cannabis use levels and SDS scores were controlled for as covariates. CWS functional impairment data was normalized with a square root transformation (as the data had a long positive tail).

### Exploratory Aim 3: What factors predict time to relapse?

A Generalised Linear Model was used to analyse the time taken to relapse (in days) (dependent variable), with the average change in functional impairment scores between baseline and abstinence as the independent variable. The analysis controlled for age, gender, SDS scores and the mean weekly cannabis use prior to entering the study.

All analyses were carried out using SPSS version 20.

## Results

**Figure 1:**
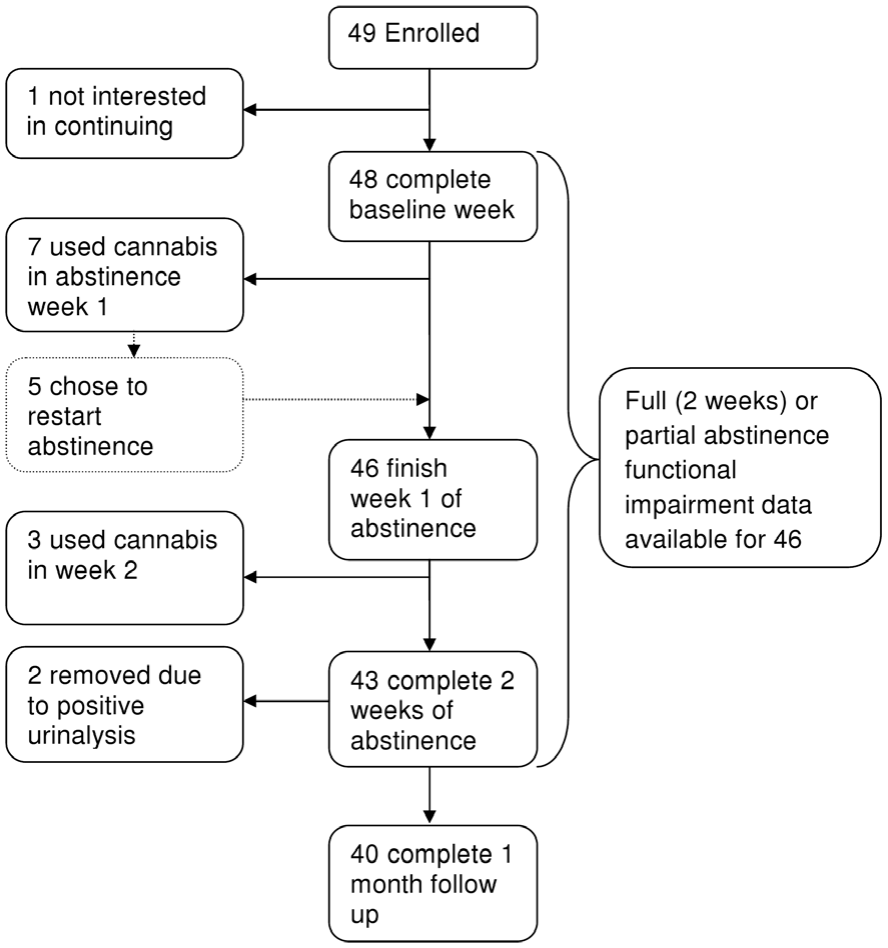
Flow chart depicting study participation.

**Figure 2:**
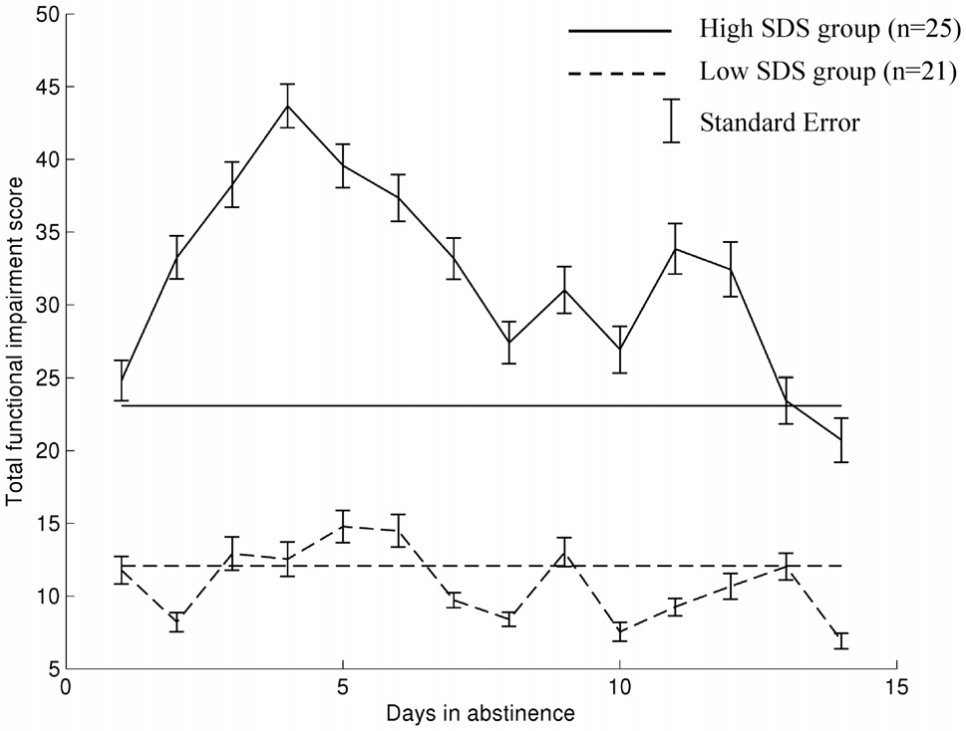
Variability of functional impairment from cannabis withdrawal over time in two subgroups formed by a “clinically informed” split in Severity of Dependence Scale scores. Total daily functional impairment scores for high and low SDS groups (horizontal lines are the average functional impairment scores rated during the baseline “smoking as usual” week for each SDS group). For the purposes of graphical demonstration, SDS group was assigned based on a clinically informed split at 8 or above for high dependent users [45].

Of the 131 people phone screened forty-nine enrolled in the study (see Table 2 for demographics and substance use information), one dropped out during baseline, and two dropped out at the start of abstinence week 1 without providing any abstinence data (Figure 1). Whilst a small proportion of study participants were diagnosed with current or past alcohol or other substance use disorders; see prior report for a full exposition of other psychiatric diagnoses [11]), all were considered mild dependencies that would not interfere with study participation. The comparison of functional impairment between those who did and did not use cannabis during the abstinence period was carried out on the remaining 46 participants who provided full or partial abstinence data. Ten people used cannabis during the attempted abstinence phase after an average of five days in abstinence (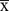 5.1, SD 3.1, range: 1–10; Figure 1), which coincided with peak functional impairment (Figure 2).

**Table 2:** Demographics and substance use.

Variable	Dependent users (N = 49)
Gender (% Male)	67
Age (years)	30 (Range: 18–57; SD 9.59)
Age of first cannabis use (years)	16.1 (Range: 11–28; SD 3.16)
Age of transition to regular cannabis use (years)	19.6 (Range: 14–40; SD 4.97)
Cannabis Severity of Dependence Scale Score	7.7 (Range: 3–15; SD 3.03)
# SCID cannabis dependence criteria endorsed	5.62 (Range: 3–7; SD 1.05)
Amount of cannabis consumed in baseline ‘smoking as usual’ week (in grams)	7.76 (Range: 1.04–43.86; SD 9.29)
# days abstinent from cannabis in the previous 3 months	0.45 (Range: 0–5; SD 0.94)
Amount of cannabis consumed per week during the one month follow up period (in grams)	2.81 grams (Range: 0–17, SD 3.1)
# Cigarettes consumed per week in the baseline ‘smoking as usual’ week	40.3 (Range: 0–150, SD 47.7)
# Cigarettes consumed per week during the two weeks of cannabis abstinence	54.02 (Range: 0–192, SD 57.4)

### Predicting withdrawal related functional impairment

The results of the model to identify factors predicting levels of functional impairment from cannabis withdrawal are listed in Table 3. The null model consists of only the intercept, and demonstrates significant heterogeneity in functional impairment scores between study participants. The addition of time in abstinence at step 1 was a significant predictor of functional impairment, explaining 8.9% of the variance. Neither of the demographic variables were significant in step 2, increasing explained variance by only 0.0008%. The addition of cannabis related variables in step 3 increased explained variance to 14%, and inspection of the univariate statistics in the full model show that only the severity of dependence (SDS scores) contributed to this increased predictive power in the model (Table 3; Figure 2).

**Table 3:** Summary of hierarchical repeated measures mixed model analysis of factors predicting functional impairment from cannabis withdrawal.

Variable	F-value(_Degrees of Freedom_)	p-value	AIC^a^	Δ*R* ^2^ *_LR_*	*R* ^2^ *_LR_* b
Total daily functional impairment score					
Null model			6767.04		0
Intercept	32.45_ (1,41.99)_	0.0001			
Step 1			6686.83	0.089	0.089
Time in abstinence	2.35_(14,365.01)_	0.004			
Step 2			6679.71	0.0008	0.097
Age	0.69_(1/40.01)_	0.41			
Gender	0.19_(1,40.11)_	0.66			
Step 3			6638.25	0.04	0.14
Cannabis Use (g/week)	0.81_(1/33.98)_	0.38			
SDS Score	4.95_(1/34.39)_	0.03			
Step 4			6164.36	0.37	0.51
Total severity of cannabis withdrawal	671.16_(1/776.907)_	0.0001			

a . Akaikes Information Criterion,

b . Likelihood ratio based R^2^ approximation.

### Relationship between withdrawal severity and associated impairment

Adding withdrawal severity scores at step 4 of the “predictors of functional impairment” model (Table 3) causes a large jump in explained variance to 51%, demonstrating a strong relationship between withdrawal severity and the functional impairment caused by withdrawal (Table 3).

### Withdrawal related functional impairment and relapse

Univariate statistics comparing the effects of functional impairment from each withdrawal symptom on chances of relapse are shown in Table 4 (high SDS) and Table 5 (low SDS). Withdrawal symptoms causing increased levels of functional impairment for people who relapsed are towards the top of the tables. Despite both high and low SDS groups having an equal number of people who relapsed (n = 5 in each group), only the high SDS group had significantly elevated functional impairment from cannabis withdrawal in those who relapsed (Tables 4 and 5). In the high SDS group, a significant interaction effect between time (baseline vs. abstinence) and relapse group was observed in seven cannabis withdrawal symptoms (I had trouble getting to sleep (F_1,23_ = 8.38, p = 0.008), I had no appetite (F_1,23_ = 7.95, p = 0.01), I felt anxious (F_1,23_ = 7.93, p = 0.01), Life felt like an uphill struggle (F_1,23_ = 7.04, p = 0.01), I felt physically tense (F_1,23_ = 5.29, p = 0.03), I had mood swings (F_1,23_ = 4.84, p = 0.04) and I felt depressed (F_1,23_ = 4.49, p = 0.05); see Table 4).

**Table 4:** Which withdrawal symptoms are associated with relapse in High Severity of Dependence Scale score users?

Withdrawal Symptom	No relapse (n = 20)		Relapse (n = 5)		Time (within subjects)		Relapse vs no relapse (between subjects)		Time×relapse (interaction)	
	BaselineMedian (IQR)	Abstinence Week 1 Median (IQR)	Baseline Median (IQR)	Abstinence Week 1 Median (IQR)	F_(1,23)_	P	F_(1,23)_	P	F_(1,23)_	P
I had trouble getting sleep	1 (2)	1.5 (4)	0 (4.5)	5 (5.5)	3.72	0.07	0.43	0.51	8.38	0.008
I had no appetite	0 (3.25)	1 (3)	1 (3.5)	5 (0.75)	3.53	0.07	2.5	0.13	7.95	0.01
I felt anxious	1 (3.75)	2 (5)	0 (3)	2.5 (6.75)	3.52	0.07	0.03	0.87	7.93	0.01
Life felt like an uphill struggle	1 (2.75)	2 (3.75)	0 (3.75)	3 (6)	3.13	0.09	0.05	0.83	7.04	0.01
I felt physically tense	1 (2)	2 (3)	1.5 (2.5)	4.5 (5.5)	2.35	0.14	1.21	0.28	5.29	0.03
I had mood swings	1 (2)	1 (4)	1 (0.34)	3.5 (4.75)	2.15	0.16	0.59	0.45	4.84	0.04
I felt depressed	0.5 (1)	1 (3)	0 (3.75)	3 (6)	1.99	0.17	0.1	0.75	4.49	0.05
I felt nauseous (like vomiting)	0 (0)	0 (1)	0.83 (0.36)	0.57 (4.7)	1.33	0.26	0.003	0.96	2.98	0.09
Total CWS functional impairment score	16 (31.5)	24 (46.75)	24.5 (13.75)	60 (62)	0.91	0.35	2.04	0.17	2.04	0.17
I yawned a lot	0 (1)	0 (2)	1 (2.25)	1.5 (4.75)	0.87	0.36	2.62	0.12	1.96	0.18
I was easily irritated	1 (2.75)	1.5 (4)	1.5 (3.25)	5 (5.2)	0.83	0.37	2.76	0.11	1.88	0.19
I felt nervous	0.5 (2)	1 (3.75)	0 (3)	1 (6)	0.8	0.38	0.002	0.97	1.81	0.19
I woke up early	2 (3.75)	1 (4)	1 (3.5)	2 (1.5)	0.68	0.42	0.03	0.86	1.53	0.23
I felt worried	1 (2)	2 (3)	0 (3)	1.5 (7.5)	0.65	0.43	0.13	0.72	1.45	0.24
Thinking about smoking	1 (3.75)	2.5 (4.5)	3.5 (4.5)	6.5 (5.5)	0.61	0.44	4.06	0.06	1.37	0.25
I had some angry outbursts	0 (2.75)	1 (2.75)	1 (0.75)	3.5 (4.75)	0.16	0.69	2.16	0.16	0.36	0.55
I had a stomach ache	0 (0)	0 (0.75)	0 (0.75)	0 (2.25)	0.14	0.72	0.008	0.93	0.3	0.59
I felt tired	2 (2.75)	2 (2.75)	3 (2.25)	3 (3.5)	0.13	0.73	1.9	0.19	0.29	0.59
Imagining being stoned	0 (2)	2 (4)	2.5 (4.75)	6 (7.25)	0.13	0.73	3.08	0.09	0.29	0.59
I had hot flashes	0 (0)	0 (2.5)	0 (0.75)	0 (1.5)	1.35	0.74	0.04	0.85	0.26	0.62
I had nightmares or strange dreams	0 (0.75)	0 (1)	0 (0.12)	0.2 (2.35)	0.03	0.86	0.9	0.35	0.08	0.79
I had a headache	0.5 (2)	1 (1.75)	0.5 (1.75)	0.5 (4)	0.007	0.94	0.05	0.83	0.02	0.91
I woke up sweating at night	0 (0.75)	1 (3.5)	0.5 (4.75)	3 (6.25)	0.009	0.93	1.45	0.23	0.02	0.88
I felt restless	1 (2.75)	2 (3)	3.5 (2.5)	5 (6)	0.007	0.94	3.61	0.07	0.02	0.9

Medians and Interquartile range (IQR) with results from a non parametric two way repeated measures ANOVA (using ranked data) comparing change in functional impairment between baseline and abstinence week 1, between people who relapsed to cannabis use during the two weeks of attempted abstinence and those who didn't. rANOVA results are presented for the main effect of relapse group, the main effect of time, and the interaction of time and relapse group. The table is sorted by the F-value from the rANOVA interaction result to reflect the relative order of withdrawal symptoms with respect to their association with relapse (e.g. items at the top of the table are those that are most associated with relapse when comparing functional impairment changes from baseline to week one of abstinence).

**Table 5:** Which withdrawal symptoms are associated with relapse in Low Severity of Dependence Scale score users?

Withdrawal Symptom	No relapse (n = 16)		Relapse (n = 5)		Time (within subjects)		Relapse vs no relapse (between subjects)		Time×relapse (interaction)	
	BaselineMedian (IQR)	Abstinence Week 1 Median (IQR)	Baseline Median (IQR)	Abstinence Week 1 Median (IQR)	F_(1,19)_	P	F_(1,19)_	P	F_(1,19)_	P
I had mood swings	0 (1)	0.5 (2)	0 (1)	0 (0.5)	0.57	0.46	0.59	0.45	2.08	0.17
I had hot flashes	0 (0)	0 (0)	0 (0)	0 (0)	0	1	0	1	1.65	0.22
I felt restless	0 (2)	0.5 (1)	1 (3)	0 (2)	0.43	0.52	0.07	0.79	1.56	0.23
I woke up early	0 (1.75)	0 (1)	0 (2)	0 (0.5)	0.3	0.59	0.28	0.61	1.08	0.31
Imagining being stoned	0.5 (2)	0 (1)	0 (1)	0 (2.5)	0.28	0.6	0.21	0.65	1.04	0.32
I was easily irritated	0 (1)	1 (2)	1 (1.5)	0 (2)	0.24	0.63	0.06	0.81	0.87	0.36
I had trouble getting sleep	0.5 (1)	1 (2)	0 (3)	0 (2)	0.22	0.65	0.22	0.65	0.79	0.39
I had some angry outbursts	0 (1)	1 (1.7)	1 (1.5)	0 (2.5)	0.2	0.66	0.15	0.7	0.73	0.41
I felt anxious	0 (2)	0 (1)	0 (1)	0 (1.5)	0.15	0.7	0.01	0.94	0.54	0.47
Thinking about smoking	1 (2)	1 (2)	0 (1.5)	0 (2.5)	1.29	0.72	0.27	0.61	0.47	0.5
I had a stomach ache	0 (1)	0 (1)	0 (0)	0 (0.5)	0.12	0.73	2.64	0.12	0.45	0.51
I woke up sweating at night	0 (0)	0 (0)	0 (0.5)	0 (1.5)	0.11	0.74	0.33	0.58	0.41	0.53
I felt nervous	0 (0.75)	0 (1)	0 (0.5)	0 (1)	0.1	0.75	0.01	0.91	0.38	0.55
Total CWS functional impairment score	6 (11)	12.5 (16)	13 (15)	8 (18)	0.1	0.75	0.2	0.66	0.37	0.55
I had a headache	0 (2.75)	1 (1)	0 (0.5)	0 (0.5)	0.1	0.76	2.08	0.17	0.35	0.56
I yawned a lot	0 (1.75)	0 (0)	0 (1)	0 (1)	0.08	0.78	0.01	0.91	0.29	0.6
I had no appetite	0 (1)	0 (0.75)	0 (2)	0 (1)	0.07	0.8	0	0.95	0.24	0.63
I felt depressed	0 (1)	0 (1)	0 (0.21)	0 (0.08)	0.06	0.81	3.92	0.06	0.23	0.64
Life felt like an uphill struggle	0 (1.75)	0 (2)	0 (0.5)	0 (0.5)	0.03	0.87	0.82	0.38	0.1	0.75
I felt worried	0 (1)	0.5 (1)	0 (1)	0 (2)	0.02	0.89	0.2	0.66	0.07	0.79
I had nightmares or strange dreams	0 0.75)	0 (0)	0 (0.5)	0 (0.5)	0.02	0.9	0.03	0.87	0.06	0.8
I felt tired	2 (2.75)	1.5 (3)	2 (3)	1 (2)	0.02	0.9	1.26	0.28	0.05	0.82
I felt physically tense	0 (1)	0 (1)	0 (1)	0 (1.5)	0.01	0.94	0	0.98	0.02	0.89
I felt nauseous (like vomiting)	0 (0)	0 (0.75)	0 (0)	0 (0.36)	0.01	0.94	0.46	0.51	0.02	0.88

Medians and Interquartile range (IQR) with results from a non parametric two way repeated measures ANOVA (using ranked data) comparing change in functional impairment between baseline and abstinence week 1, between people who relapsed to cannabis use during the two weeks of attempted abstinence and those who didn't. rANOVA results are presented for the main effect of relapse group, the main effect of time, and the interaction of time and relapse group. The table is sorted by the F-value from the rANOVA interaction result to reflect the relative order of withdrawal symptoms with respect to their association with relapse (e.g. items at the top of the table are those that are most associated with relapse when comparing functional impairment changes from baseline to week one of abstinence).

The multivariate analysis used a logistic regression to test whether the withdrawal symptoms identified (above) as causing significant functional impairment in high SDS relapsers were predictive of relapse for the group as a whole. The omnibus tests in Table 6 show that increased somatic withdrawal symptoms are predictive of relapse, and that within the somatic symptoms multivariate model, only increased physical tension is a significant predictor of relapse. Neither the negative affect model nor the combined negative affect and somatic symptoms model had significant omnibus tests for predictors of relapse, although within the combined model, physical tension remained the only significant predictor of relapse, with every one point increase above baseline physical tension levels leading to a four times increase in the proportional odds of relapsing.

**Table 6:** Best fitting multivariate models using somatic variables only, negative affect variables only, and then the combination somatic and negative affect variables to predict relapse during the attempted abstinence.

Withdrawal measure	Wald/Omnibus Chi Sq	*p* value	OR	OR 95% CI
**Somatic variables only model**	8.525	0.036		
I had trouble getting to sleep (abstinence – baseline)	1.71	0.19	0.59	0.27, 1.29
I had no appetite (abstinence – baseline)	0.93	0.33	1.38	0.71, 2.69
I felt physically tense (abstinence – baseline)	4.4	0.036	2.52	1.06, 5.99
**Negative affect variables only model**	3.03	0.55		
I felt anxious (abstinence – baseline)	0.76	0.38	1.36	0.68, 2.75
Life felt like an uphill struggle (abstinence – baseline)	0.03	0.87	1.08	0.42, 2.82
I had mood swings (abstinence – baseline)	0.24	0.62	0.8	0.33, 1.93
I felt depressed (abstinence – baseline)	0.35	0.55	1.38	0.47, 4.11
**Combined somatic and negative affect model**	10.82	0.15		
I had trouble getting to sleep (abstinence – baseline)	1.98	0.16	0.58	0.27, 1.24
I had no appetite (abstinence – baseline)	0.96	0.33	1.44	0.69, 3
I felt physically tense (abstinence – baseline)	4.77	0.02	3.74	1.14, 12.19
I felt anxious (abstinence – baseline)	0.05	0.83	1.1	0.46, 2.6
Life felt like an uphill struggle (abstinence – baseline)	0.59	0.44	1.6	0.47, 5.7
I had mood swings (abstinence – baseline)	1.7	0.19	0.47	0.15, 1.5
I felt depressed (abstinence – baseline)	0.087	0.77	0.81	0.21, 3.2

As sample sizes are small in the relapse groups, a *post hoc* analysis based on the pre-post changes in Total CWS Functional Impairment Scores between relapsers and non relapsers from both SDS groups combined (No relapse: Baseline mean (SD) 19.07 (24.5), Abstinence week 1 mean (SD) 26.19 (29.72); Relapse: Baseline mean (SD) 16.45 (11.53), Abstinence week 1 mean (SD) 33.47 (35.36)) suggests that an effect size (Cohens *f*) of 0.18 was observed. When used in a between factors mixed ANOVA power analysis (with 2 groups and 2 measurements), with α = 0.05 and a minimum power of 80%, this effect size suggests that a total sample size of 64 (32 in each group) would be required to detect an effect of this magnitude between people who relapse and those who do not.

The time to relapse variable was normally distributed amongst those who relapsed (Shapiro-Wilk (9) = 0.96, p = 0.8) but was not so when the full sample was considered (i.e. including people who succeeded in remaining abstinent for the full 14 days; Shapiro-Wilk (45) = 0.52, p = 0.0001). There was no difference in any of the following variables between those who relapsed and those who didn't: age (F_1,45_ = 1.69, p = 0.2, gender (Fishers exact test, p = 0.69), endorsement of SCID withdrawal dependence criteria (Fishers exact test, p = 0.7), the number of SCID dependence criteria endorsed (Pearson Chi Square = 0.83, p = 0.9), SDS score (F_1,45_ = 0.005, p = 0.94), or the amount of cannabis they smoked prior to entering the study (F_1,45_ = 0.031, p = 0.86). Functional impairment scores did not predict the number of days taken to relapse when controlling for age, gender, SDS score and pre-study cannabis use (F_5, 41_ = 0.56, p = 0.7).

### The effects of functional impairment during abstinence on cannabis use at follow up

To assess the relationship between functional impairment during abstinence and cannabis use during the one-month follow-up data were available for 40 of the 46 study participants. The six participants lost to follow up experienced significantly greater functional impairment from their withdrawal symptoms during the first week of abstinence (relative to baseline) than those who were followed up (week 1 mean total functional impairment increase for those lost to follow up = 26.8, mean increase for those who were able to be followed up = 6.8; F_1, 45_ = 6.6, p = 0.01), however the two groups of participants did not differ in their functional impairment scores during week 2 of abstinence (F_1, 45_ = 0.001, p = 0.97). Those lost to follow up were more likely to have relapsed: 40% (n = 4) of those who relapsed (n = 10) could not be followed up compared to only 5% (n = 2) of those who were able to maintain abstinence for the full two week period (n = 36) (Fishers exact test, p = 0.014). Those lost to follow up did not differ from the remaining 40 study participants in any of the following variables: age (F_1,45_ = 0.39, p = 0.53), gender (Fishers exact test, p = 0.65), SDS Score (F_1, 45_ = 0.48, p = 0.49), or the amount of cannabis they consumed prior to entering the study (F_1,45_ = 0.06, p = 0.8). Higher levels of functional impairment during the abstinence period significantly predicted higher levels of self-reported cannabis use at 1 month follow up (β = 0.019 (SE 0.39), t = 4.197, p = 0.0001), after controlling for baseline cannabis use levels (β = 0.025 (SE 0.01), t = 2.185, p = 0.029) and SDS scores (β = −0.19 (SE 0.06), t = −3.3, p = 0.001).

## Discussion

Consistent with previous work on withdrawal severity [11], higher levels of dependence on cannabis were associated with higher levels of functional impairment from cannabis withdrawal (Table 3 and Figure 2). The strongest predictor of functional impairment to normal daily activities from cannabis withdrawal was the severity of the cannabis withdrawal symptoms (Table 3). As tobacco use increased during abstinence compared to the baseline ‘smoking as usual’ week (Table 2), it is unlikely that the observed impairment was due to nicotine withdrawal. Relapse to cannabis use was associated with higher levels of functional impairment in the high SDS user group (Table 4). Despite the fact that members of the low SDS group also relapsed during the abstinence attempt, their relapse was not associated with significant levels of functional impairment from withdrawal (Table 5).

Whilst the univariate analysis showed a subset of withdrawal symptoms were associated with increased functional impairment in those who relapsed (I had trouble getting to sleep, I had no appetite, I felt anxious, Life felt like an uphill struggle, I felt physically tense, I had mood swings and I felt depressed; Table 4), the multivariate predictive model indicated that only “physical tension” remained a significant predictor of relapse for the whole group (Table 6). These findings may suggest that somatic and negative affect symptoms respond similarly during a quit attempt, but somatic withdrawal symptoms may be more pertinent to predicting relapse in this sample of non-treatment seekers. If the same were observed in a clinical sample, this may be useful for counselling cannabis smokers on what changes to expect during their quit attempt. However it is important to stress that the multivariate models may suggest which withdrawal symptoms integrate relapse risk information efficiently, rather than revealing specific causal paths.

It is of note that the average level of functional impairment caused by cannabis withdrawal symptoms was relatively mild (the highest median total CWS functional impairment scores during abstinence were 60 out of a possible 190 during week 1 of abstinence in high SDS users who relapsed; Table 4) among this sample of non-treatment seeking users. Several factors should be considered when interpreting these data. First, the data represent only one aspect of the withdrawal syndrome – that being cannabis users' perception of the impact of withdrawal symptoms on carrying out their normal daily activities. Functional impairment received a uniformly lower endorsement for all of the symptoms surveyed relative to symptom intensity scores [11]. Second, the focus on average values across all of the participants in the study masks the variation in functional impairment experienced between people. As can be seen from the ‘interquartile range’ data presented in Tables 4 and 5, some study participants reported that cannabis withdrawal symptoms caused very high levels of functional impairment. Third, the study population consisted of non-treatment seekers, so it is reasonable to expect that higher levels of withdrawal-related functional impairment would be reported by treatment seekers, and this will be a fruitful avenue for future research. Finally, whilst the cannabis withdrawal syndrome is mild for most users, it appears comparable with tobacco withdrawal [20], [24], [46] which is of well established clinical significance.

This study has several notable limitations. The sample size is small, which can lead to inflated Type I errors in the analyses, and precludes conduct of factor analyses on the CWS to test any *a priori* predictions on the underlying structure of the cannabis withdrawal syndrome. The *ad hoc* analyses grouping selected symptoms into somatic and negative affect variables used in this present work would benefit from more rigorous factor analytical methods with larger sample sizes. The relapse analysis was by necessity opportunistic (hence we did not set out a formal *a priori* power calculation for this analysis), and the small numbers of participants in the relapse group suggest that any findings relating to relapse would benefit from further research. The *post-hoc* power analysis of total withdrawal scores suggests that ∼64 participants (32 in each group), would be required to detect a difference of the magnitude observed in this study. However effect size calculation from small sample sizes is prone to error [47], further supporting the need to follow up the relapse findings with larger datasets. As mentioned previously, a clear limitation of the present findings is that the study population was generally non-treatment seeking, so it may represent a conservative account of the findings in a treatment delivery context. Performing the same study in a clinical treatment seeking group may be expected to find more severe withdrawal having greater negative consequences to daily life, with the potential for greater levels of relapse. Examining withdrawal in such clinical samples will be a fruitful area of future research. It is also worthy of note that Table 4 shows measureable levels of functional impairment at baseline (before abstinence from cannabis) for the high SDS group. This is consistent with previous studies of both cannabis [48] and tobacco [46] withdrawal, and is expected as each individual will have their own baseline level of functioning, for example a mild usual sleep problem or a usual mildly depressed mood, which may become more substantial during abstinence. Finally there was no external corroboration of the self-reported functional impairment, or use of an alternative functional impairment measure.

In conclusion, cannabis withdrawal is clinically significant because it is associated with elevated functional impairment to normal daily activities, and the more severe the withdrawal is, the more severe the functional impairment is. Elevated functional impairment from a cluster of cannabis withdrawal symptoms is associated with relapse in more severely dependent users. Those participants with higher levels of functional impairment from cannabis withdrawal also consumed more cannabis in the month following the end of the experimental abstinence period. Higher levels of cannabis dependence (scores on the SDS) predicted greater functional impairment from cannabis withdrawal. These findings suggest that higher SDS scores can be used to predict problematic withdrawal requiring more intense treatment that can be monitored closely using the Cannabis Withdrawal Scale (Table 1) [11]. Finally and speculatively, the finding that lower levels of cannabis dependence predict lower levels of functional impairment from withdrawal (and thus lower levels of relapse) may indicate that stepped reductions in cannabis use prior to a quit attempt could reduce dependence, and thus reduce levels of withdrawal related functional impairment, improving chances of achieving and maintaining abstinence. Targeting the withdrawal symptoms that contribute most to functional impairment during a quit attempt might be a useful treatment approach (e.g. stress management techniques to relieve physical tension and possible pharmacological interventions for alleviating the physical aspects of withdrawal such as loss of appetite and sleep dysregulation).
